# Role and Utility of Mixed Reality Technology in Laparoscopic Partial Nephrectomy: Outcomes of a Prospective RCT Using an Indigenously Developed Software

**DOI:** 10.1155/2022/8992051

**Published:** 2022-05-16

**Authors:** Nariman Gadzhiev, Igor Semeniakin, Aleksandr Morshnev, Antonio Alcaraz, Vineet Gauhar, Zhamshid Okhunov

**Affiliations:** ^1^Department of Urology, Saint Petersburg State University Hospital, Saint Petersburg, Russia; ^2^Department of Urology, A. I. Yevdokimov Moscow State University of Medicine and Dentistry, Moscow, Russia; ^3^Bauman Moscow State Technical University, Moscow, Russia; ^4^Department of Urology, Hospital Clinic, Barcelona, Spain; ^5^Department of Urology, Ng Teng Fong General Hospital (NTFGH), Singapore; ^6^Department of Urology, University of California, Irvine, CA, USA

## Abstract

**Objective:**

To develop a software for mixed reality (MR) anatomical model creation and study its intraoperative clinical utility to facilitate laparoscopic partial nephrectomy.

**Materials and Methods:**

After institutional review board approval, 47 patients were prospectively randomized for LPN into two groups: the control group (24 patients) underwent operation with an intraoperative ultrasound (US) control and the experimental group (23 patients) with smart glasses HoloLens 2 (Microsoft, Seattle, WA, USA). Our team has developed an open-source software package called “HLOIA,” utilization of which allowed to create and use during surgery the MR anatomical model of the kidney with its vascular pedicle and tumor. The study period extended from June 2020 to February 2021 where demographic, perioperative, and pathological data were collected for all qualifying patients. The objective was to assess the utility of a MR model during LPN and through a 5-point Likert scale questionnaire, completed by the surgeon, immediately after LPN. Patient characteristics were tested using the chi-square test for categorical variables and Student's *t*-test or Mann–Whitney test for continuous variables.

**Results:**

Comparison of the variables between the groups revealed statistically significant differences only in the following parameters: the time for renal pedicle exposure and the time from the renal pedicle to the detection of tumor localization (*p* < 0.001), which were in favor of the experimental group. The surgeon's impression of the utility of the MR model by the proposed questionnaire demonstrated high scores in all statements.

**Conclusions:**

Developed open-source software “HLOIA” allowed to create the mixed reality anatomical model by operating urologist which is when used with smart glasses has shown improvement in terms of time for renal pedicle exposure and time for renal tumor identification without compromising safety.

## 1. Introduction

Laparoscopic partial nephrectomy (LPN) is the standard of care for clinical T1 renal tumors [1–3] and can be a technically challenging procedure with a steep learning curve [4–8]. The critical steps during LPN include renal pedicle exposure [9], identification of the renal tumor, complete excision, and renorrhaphy [10]. As most minimally invasive procedures lack direct visual inspection of the surgical area, we rely on imaging such as computer tomography (CT) or magnetic resonance imaging (MRI). Poor preoperative planning and negligence during the exposure of the renal pedicle increases the risk of vascular injury and may lead to a significant bleeding often hard to control and requiring a conversion to an open surgery [11]. In turn, a thorough preoperative planning facilitates precise intraoperative identification of the tumor and its relationship with surrounding structures allowing for precision-based resection, thereby, minimizing complications and positive surgical margins whilst maximizing clinical outcomes, especially in complex renal tumors [12].

Over the decades, various technological innovations have been developed to improve oncological outcomes in laparoscopic and robotic surgery, particularly focused at enhanced intraoperative tumor visualization such as 3D laparoscopic surgery, use of artificial intelligence (AI) based software, deep learning 3D model reconstructions, augmented reality (AR), virtual reality (VR), and mixed reality (MR). The latter is an emerging technology different from virtual and augmented realities as herein it overlays virtual objects that can be manipulated whilst still being anchored to the physical environment [13]. Usually, creation of AR and MR anatomical models needs dedicated staff such as bioengineers and graphic designers, which may be a real obstacle to the widespread use of these technologies.

The primary goal of our study was to indigenously develop a software that allows to create and utilize the MR anatomical model and study its clinical utility to help improve LPN outcomes. The secondary goal of the study was to evaluate the subjective utility of the MR model as an intraoperative reference tool.

## 2. Methods

### 2.1. Study Design and Randomization

**Figure 1:**
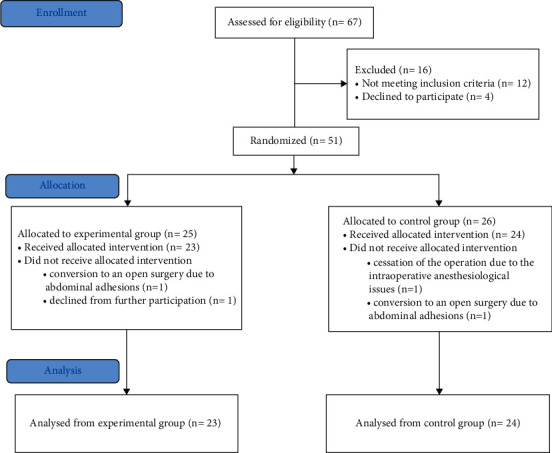
Flow of participants in the “mixed reality technology for LPN” study.

After institutional review board approval, we performed a prospective randomized clinical trial to enroll patients with T1a renal tumors planned for LPN between June 2020 and February 2021. Patients were randomized into two groups. Group 1, the control group, consisted of patients who underwent LPN with solely intraoperative laparoscopic ultrasonography (US). Group 2 consisted of patients who underwent LPN facilitated with the MR model (Figure 1). Randomization was performed using a computerized randomization program (Jamovi 1.8.1, Randomizer Module, Sydney, Australia) and sealed envelopes.

Inclusion criteria were as follows: adults 18 years of age or older, able to sign an informed consent in full mental capacity, preoperatively diagnosed on conventional imaging (CT and or USG) with T1 small renal mass, and amenable for LPN. For each patient, we prospectively collected demographic data including age, body mass index, clinical tumor size, tumor side, location, and complexity score according to the PADUA scoring system; perioperative data, including time for renal pedicle exposure (from the moment of lower pole exposure to the renal pedicle) and time for renal tumor detection (from the moment of Gerota's fascia incision to the detection of renal tumor); pathological data; and data on postoperative functional outcome and complications, classified according to the Clavien–Dindo system. Exclusion criteria were as follows: patients unwilling to participate or did not meet the inclusion criteria.

### 2.2. Preparation of the Mixed Reality Model

**Figure 2:**
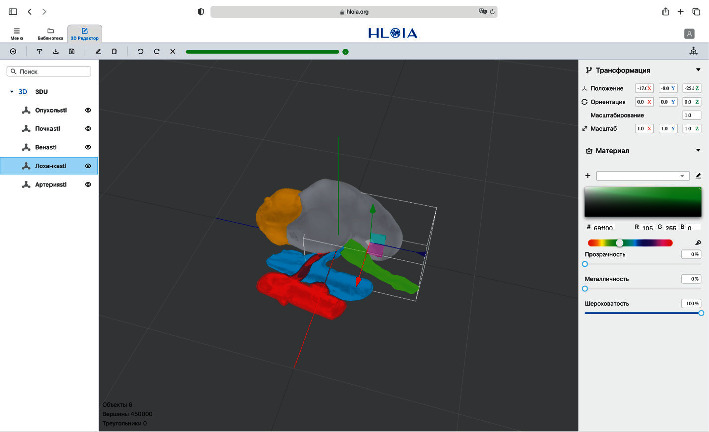
HLOIA's web application 3D editor desktop.

All patients included in the study underwent enhanced multidetector computed tomography (CT) preoperatively with 0.5 mm thin slice images in a lateral decubitus position. The position during scanning is close to the position of the patient undergoing LPN to minimize inner organ displacement. DICOM images were processed by the “Inobitec DICOM Viewer Pro” (Voronezh, Russia) software. To create a 3D model, the images were segmented by the renal vasculature (both arterial and venous), tumor, kidney surface, and collecting system using either the dynamic region growing or the watershed method. The 3D model created was exported as a stereolithography (STL) file. To obviate the necessity for additional staff like bioengineers and graphic designers, we have developed a software package called “HLOIA” which stands for “Healthy Life: Operations with Innovative Assistance” and is open-source. HLOIA consists of three distinct parts: a web application, a cloud server, and a client application for smart glasses. Previously prepared STL files were uploaded by the surgeon to the HLOIA web application. The HLOIA web application is based on the Three.js library for working with 3D objects and is located at https://hloia.org/. In the Editor section of the HLOIA web application, color and transparency settings of the MR model were adjusted (Figure 2) and saved to the HLOIA cloud server, which is based on .NET Core 3.1.

For the next step, the surgeon must have high-speed wireless internet access and HoloLens 2 smart glasses (Microsoft, Seattle, WA, USA). The surgeon must access the HLOIA website to install the HLOIA client on the glasses. The HLOIA client was created using the Unity platform and Mixed Reality Toolkit for Unity.

**Figure 3:**
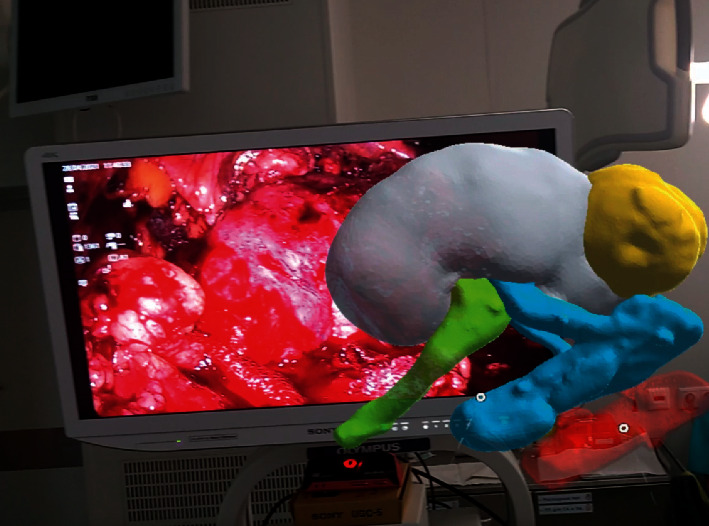
Transparent 3D mixed reality model anchored in front of the surgeon and matched to the real-time videoendoscopic picture during LPN.

After authorization in the application, the previously saved MR model on a cloud server is downloaded and then becomes available for offline use. The obtained MR model could be anchored to any point in the physical world, and the wearer could view the model from any position (Figure 3). Resizing and rotation of the MR model is possible using hand gesture commands.

The entire process of MR model creation from CT segmentation up to downloading it to the smart glasses from the cloud server takes approximately 20 minutes on average and is performed solely by the operating urologist without previous experience in bioengineering or graphic design.

### 2.3. Intervention

All patients underwent LPN via the transperitoneal approach in the standard lateral decubitus position by a single surgeon with experience in more than 300 laparoscopic surgeries. In the control group, for the study purposes, the first step was exposure of the lower pole. The next step was renal pedicle exposure. Gerota's (anterior renal) fascia was then opened to locate the tumor. The fat over the tumor was conserved if possible. After detecting the tumor, the decision to clamp the renal pedicle or not was based on the surgeon's discretion. Using both resection and enucleation techniques, the tumor was removed. After tumor removal, the tumor bed was closed in a watertight manner with a 3-0 barbed suture.

In the experimental group, the steps were as follows. Step 1: exposure of the lower pole of the kidney. Step 2, lower pole of the kidney in the mixed reality model was manually aligned to that of the patient's kidney. This step is critical for anatomical matching to demonstrate the exact location of the renal pedicle and tumor location under the guidance of the superimposed MR kidney model. Step 3, the location of the tumor was verified using an ultrasound probe in every case. After visual and spatial mapping of the renal tumor, the MR model was removed from the videoendoscopic picture and used as a reference tool during the rest of the surgery.

### 2.4. Questionnaire

After each LPN in the experimental group, the surgeon completed a 5-point Likert scale questionnaire for the subjective assessment of the MR model utility during LPN. The questionnaire included three items rated on a 5-point Likert scale: 1, strongly disagree; 2, disagree; 3, not clear; 4, agree; and 5, strongly agree. The statements were as follows:

1. “The 3D holographic kidney model was useful for renal pedicle exposure.”
2. “The 3D holographic kidney model was useful for locating renal tumor.”
3. “The 3D holographic kidney model was useful as a reference tool during LPN.”

### 2.5. Statistics

Patient characteristics were tested using the chi-square test for categorical variables and Student's *t*-test or Mann–Whitney test for continuous variables. All results for continuous variables are expressed as mean (SD) or median (IQR), and frequencies and proportions were reported as percentages. Intraoperative and postoperative variables were evaluated, and the differences in quantitative and categorical variables were tested using nonparametric Mann–Whitney and chi-square tests, respectively. Quantitative variables were illustrated with box-and-whisker plots and frequency histograms with densities. Categorical and range variables are presented as bar plots. The questionnaire results were illustrated using a radar chart. For every comparison, exact *p* values were shown, and the results were considered statistically significant when *p* ≤ 0.05. Data collection was carried out using MS Excel 2016, and statistical processing was implemented using the software package Jamovi v.1.8.1.

## 3. Results

A total of 47 patients were randomized into the study. There was no statistical significance in preoperative values such as age, BMI, HgB, creatinine level, GFR, and tumor characteristics such as location, size, and complexity scores (Table 1).

**Figure 4:**
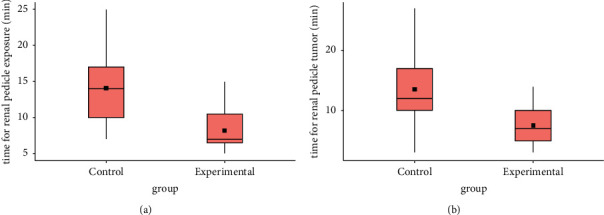
Time parameters for renal pedicle exposure (a) and for tumor location determination (b).

Comparison of intra and postoperative variables between the groups revealed statistically significant differences in the following parameters: time for renal pedicle exposure and time from renal pedicle to detection of tumor location (*p* < 0.001) in favor of the experimental group (Figure 4). For the indicated variables, large effects were also observed: Cohen's *d* = 1.36 and 1.23, respectively. Ultrasound control confirmed the presence of a tumor in 100% of the cases in the experimental group. The functional outcome evaluation based on creatinine and calculated eGFR values revealed insignificant differences between the groups 101 ± 33.8 and 88 ± 35 (*p*=0.35).

The rate of postoperative complications did not differ between the groups (*p*=0.58). For the rest of the variables, including pathological data and the rate of positive surgical margins, no statistically significant differences were found either (Table 2).

The mean score of the first statement in the questionnaire “3D holographic kidney model was useful for renal pedicle exposure” was 4.78 ± 0.42. The mean scores for the second and third statements were 4.13 ± 0.55 and 4.35 ± 0.49, respectively.

**Figure 5:**
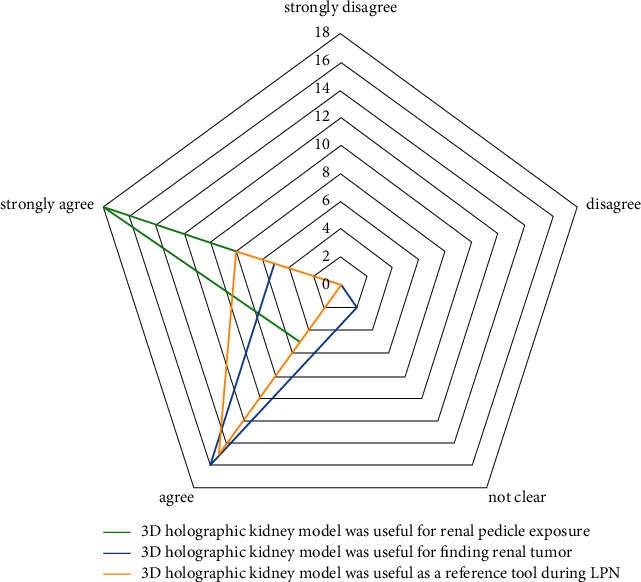
Radar chart for questionnaire answer options.

The surgeon's impression of the utility of the MR model assessed with the proposed questionnaire demonstrated high scores in all given statements (Figure 5).

## 4. Discussion

**Figure 6:**
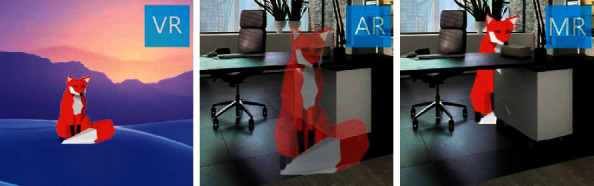
Difference between 3 main domains of immersive technology: VR is an artificial world; AR is an object of augmented reality seen on the screen; MR is a 3D object seen as a hologram which is totally controllable.

With improvements in optics, endoscopic TV monitoring systems, and surgical instruments, laparoscopic partial nephrectomy has become a viable alternative to open surgical treatment for kidney tumors up to 4 cm [14]. Preoperative enhanced computed or magnet tomography usually provides insights into the number of vessels of the renal pedicle, their route, and exact tumor location [15]. Preoperative images can be brought into the operating theater and placed on an additional screen for reference purposes. Unfortunately, this setup creates a situation in which the surgeon is forced to focus both on the surgical field and look away from the field to consult the preoperative image data on a 2-dimensional screen leading to the so called “switching focus problem” [16, 17]. Different imaging modalities such as ultrasonography, fluorescence imaging, optical coherence tomography, and ex vivo magnetic resonance imaging can be used for improved intraoperative visualization such as for renal tumor detection, but none of them can be applied uniformly for intraoperative enhancement of surgical dissection in every patient [18, 19]. A three-dimensional (3D) printed model was studied and found to be a reliable tool for preoperative planning and intraoperative navigation because it can reveal the real size, depth, and location of both the kidney mass and arteriovenous systems and may thus prevent damage to the surrounding structures [20]. Instead of using printed 3D anatomical models, production of which is costly and time-consuming, immersive technology (IT) that blurs the boundary between the physical and virtual worlds can come to the rescue. For clarity, the IT definition includes augmented reality (AR), virtual reality (VR), and mixed reality (Figure 6) [21]. Although AR utilization during partial nephrectomy has been thoroughly studied and provides clinical benefit, it requires additional staff such as software engineers for setting up and using the software and additional hardware such as a tracking system, video parsers, and powerful computers [22–24]. MR is an emerging technology that overlays virtual objects that can be manipulated and that are anchored to the physical environment. It has already been used with smart glasses as a new tool for visualization of preoperative images that facilitates anatomical understanding by the patient [25, 26]. To make the process of acquisition of the MR model easier and less demanding, we have developed a dedicated software called “HLOIA.” With its assistance, it has become possible to prepare the MR model by the operating surgeon in 20 minutes. Our study revealed statistically significant differences in time for renal pedicle exposure and time from renal pedicle to detection of tumor location (*p* < 0.001) in favor of the experimental group with large effects size: Cohen's *d* = 1.36 and 1.23, respectively. Ultrasound control confirmed the presence of a tumor in 100% of cases in the experimental group, indicating the accuracy of kidney tumor detection with the use of a superimposed MR model. When the surgeon assessed the utility of the MR model during LPN, high scores were given for all statements in the proposed questionnaire. To our knowledge, this is the first study on the intraoperative utilization of mixed reality technology for LPN.

Our study has some limitations. First, we rely on precise segmentation of the preoperative CT image of the kidney and MR model matching with real-time endoscopic picture in OR. These are done manually and are subjected to human error; however, the use of automated segmentation software to obtain more precise segmentation images and intraoperative automated MR model calibration could be a solution. Second, the sample size of our study was limited owing to the newness of this technology, yet we were able to demonstrate significant differences in the sample size enrolled prospectively. Third, even though “HLOIA” software is an open-source, one needs smart glasses for MR model use, which are relatively expensive. The retail cost of HoloLens 2 glasses (Microsoft, Seattle, WA, USA) utilized in our study is approximately four thousand US dollars.

Despite the abovementioned limitations, our findings demonstrate time savings during renal pedicle exposure and renal tumor identification in favor of the experimental group. This has given us confidence to proceed on our future projects: identification of totally endophytic masses with the MR model in comparison with ICG imaging [27] and on automated precise MR model matching with real-time endoscopic images during whole LPN in an electromagnetic field which will make utilization of MR technology more accurate and useful.

## 5. Conclusion

The utilization of mixed reality technology during laparoscopic partial nephrectomy with the use of indigenously developed software called “HLOIA” and smart glasses has shown improvement in terms of time for renal pedicle exposure and time for renal tumor identification without compromising safety and effectiveness. Our findings indicate that this technology has the potential to enhance real-time precision-based surgery.

## Figures and Tables

**Table 1:** Descriptive analysis of preoperative features.

	Control	Experimental	*P* value
Number of patients	24	23	—
Gender, no. (%)			
Male	10 (41.7)	10 (43.5)	0.90
Female	14 (58.3)	13 (56.5)	
Age (y), mean (SD)	58.7 (11.4)	58 (10.4)	0.83
BMI (kg/m^2^), mean (SD)	28.1 (5.51)	28.3 (3.65)	0.83
HB preoperative (g/l), mean (SD)	137 (10.3)	134 (10.7)	0.26
GFR preoperative (ml/min), median (IQR)	100 (56.6)	102 (35.9)	0.62
Creatinine preoperative mcmol/l, median (IQR)	68.5 (13)	76 (27.1)	0.05
Kidney face, no. (%)			
Anterior	14 (58.3)	16 (69.6)	0.42
Posterior	10 (41.7)	7 (30.4)	
Kidney rim, no. (%)			
Lateral	11 (45.8)	11 (47.8)	0.89
Medial	13 (54.2)	12 (52.2)	
Kidney pole, no. (%)			
Superior pole	4 (16.7)	9 (39.1)	0.09
Mesorenal location	12 (50)	5 (21.7)	
Inferior pole	8 (33)	9 (39.1)	
Side, no. (%)			
Left	14 (58.3)	12 (52.2)	0.67
Right	10 (41.7)	11 (47.8)	
Tumor size (mm), median (IQR)	27 (18.5)	33 (6)	0.21
PADUA score, median (IQR)	8 (3.25)	8 (2.5)	0.89

**Table 2:** Intra and postoperative variables.

	Control	Experimental	*P* value
Hb after surgery (g/l), mean (SD)	120 (17.1)	125 (14.6)	0.28
Operative time (min), mean (SD)	106 (28.4)	95 (24)	0.15
Time for renal pedicle exposure (min), mean (SD)	14.08 (5.4)	8.22 (2.76)	**<0.001**
Time from renal pedicle to tumor, min, mean (SD)	13.54 (5.99)	7.52 (3.36)	**<0.001**
Hemorrhage volume (ml), median (IQR)	150 (200)	100 (150)	0.11
Creatinine postoperative (mcmol/l), median (IQR)	101 (33.8)	88 (35)	0.35
RA clamping, no. (%)			
No	2 (8.3)	6 (26.1)	0.11
Yes	22 (91.7)	17 (73.9)	
Global ischemia time (min), mean (SD)	15.1 (7.73)	17 (7.33)	0.38
Conversion to nephrectomy, no. (%)			
No	22 (91.7)	23 (100)	0.16
Yes	2 (8.3)	0 (0)	
Clavien–Dindo score <3, no. (%)			
No	22 (91.7)	22 (95.7)	0.58
Yes	2 (8.3)	1 (4.3)	
Clavien–Dindo ≥3, no. (%)			
No	23 (95.8)	23 (100)	0.32
Yes	1 (4.2)	0 (0)	
Histopathological findings, no. (%)			
RO^*∗*^	1 (4.2)	2 (8.7)	0.78
PRCC^*∗∗*^	1 (4.2)	2 (8.7)	
ccRCC^*∗∗∗*^	20 (83.3)	18 (78.3)	
Others	2 (8.3)	1 (4.3)	
Surgical margin, no. (%)			
Positive	2 (8.3)	0 (0)	0.49
Negative	22 (91.7)	23 (100)	
GFR postoperative (ml/min), median (IQR)	65.6 (62.9)	85.2 (48.4)	0.47
US tumor control, no. (%)	—	23 (100)	—

^*∗*^Renal oncocytoma; ^*∗∗*^papillary renal cell carcinoma; ^*∗∗∗*^clear cell renal cell carcinoma.

## Data Availability

The data used to support the findings of this study are available from the corresponding author upon request.

## Abbreviations

MR: Mixed reality
LPN: Laparoscopic partial nephrectomy
US: Ultrasound
3D: Three-dimensional
CT: Computed tomography.

## References

[1] Campbell S., Uzzo R. G., Allaf M. E. (2017). Renal mass and localized renal cancer: AUA guideline. *The Journal of Urology*.

[2] You C., Du Y., Wang H. (2020). Laparoscopic versus open partial nephrectomy: a systemic review and meta-analysis of surgical, oncological, and functional outcomes. *Frontiers in Oncology*.

[3] Gong E. M., Orvieto M. A., Zorn K. C., Lucioni A., Gary D. S., Shalhav A. L. (2008). Comparison of laparoscopic and open partial nephrectomy in clinical T1a renal tumors. *Journal of Endourology*.

[4] Orvieto M. A., Chien G. W., Tolhurst S. R. (2005). Simplifying laparoscopic partial nephrectomy: technical considerations for reproducible outcomes. *Urology*.

[5] Raison N., Doeuk N., Malthouse T., Kasivisvanathan V., Lam W., Ben C. (2016). Challenging situations in partial nephrectomy. *International Journal of Surgery*.

[6] Spana G., Haber G.-P., Dulabon L. M. (2011). Complications after robotic partial nephrectomy at centers of excellence: multi-institutional analysis of 450 cases. *The Journal of Urology*.

[7] Ramani A. P., Desai M. M., Steinberg A. P. (2005). Complications of laparoscopic partial nephrectomy in 200 cases. *The Journal of Urology*.

[8] Sautter T., Haueisen H., Stierli P., Kwiatkowski M. F. (2001). A severe complication of retroperitoneoscopic nephrectomy. *The Journal of Urology*.

[9] Yang F., Zhou Q., Li X., Xing N. (2019). The methods and techniques of identifying renal pedicle vessels during retroperitoneal laparoscopic radical and partial nephrectomy. *World Journal of Surgical Oncology*.

[10] Rod X., Peyronnet B., Seisen T. (2016). Impact of ischaemia time on renal function after partial nephrectomy: a systematic review. *BJU International*.

[11] Arora S., Rogers C. (2018). Partial nephrectomy in central renal tumors. *Journal of Endourology*.

[12] Lin C.-H., Chang C. H., Chang Y. H., Lin C. H. (2020). Three-dimensional reconstruction of renal vascular tumor anatomy to facilitate accurate preoperative planning of partial nephrectomy. *Biomedicine*.

[13] Klein G., Wang H., Elshabrawy A. (2021). Analyzing national incidences and predictors of open conversion during minimally invasive partial nephrectomy for cT1 renal masses. *Journal of Endourology*.

[14] Li K. C., Wong B. T. M. (2021). A literature review of augmented reality, virtual reality, and mixed reality in language learning. *International Journal of Mobile Learning and Organisation*.

[15] Ljungberg B., Albiges L., Bedke J. (2021). Anon: EAU guidelines. https://uroweb.org/wp-content/uploads/EAU-Guidelines-on-Renal-Cell-Carcinoma-2021.pdf.

[16] Wunderlich H., Reichelt O., Schubert R., Zermann D.-H. J. (2001). Preoperative simulation of partial nephrectomy with three-dimensional computed tomography. *BJU International*.

[17] Meulstee J. W., Nijsink J., Schreurs R. (2019). Toward holographic-guided surgery. *Surgical Innovation*.

[18] Lasser M. S., Doscher M., Keehn A., Chernyak V., Evan G., Reza G. (2012). Virtual surgical planning: a novel aid to robot-assisted laparoscopic partial nephrectomy. *Journal of Endourology*.

[19] Chopra S., Bove A. M., Gill I. S., Su L. M. (2017). Robotic partial nephrectomy: advanced techniques and use of intraoperative imaging. *Atlas of Robotic Urologic Surgery*.

[20] Hekman M. C. H., Rijpkema M., Langenhuijsen J. F., Boerman O. C., Egbert O., Peter F. A. M. (2018). Intraoperative imaging techniques to support complete tumor resection in partial nephrectomy. *European Urology Focus*.

[21] Komai Y., Sugimoto M., Gotohda N. (2016). Patient-specific 3-dimensional printed kidney designed for 4D surgical navigation: a novel aid to facilitate minimally invasive off-clamp partial nephrectomy in complex tumor cases. *Urology*.

[22] Fan G., Li J., Li M. (2018). Three-dimensional physical model-assisted planning and navigation for laparoscopic partial nephrectomy in patients with endophytic renal tumors. *Scientific Reports*.

[23] Bernhardt S., Nicolau S. A., Soler L., Doignon C. (2017). The status of augmented reality in laparoscopic surgery as of 2016. *Medical Image Analysis*.

[24] Porpiglia F., Checcucci E., Amparore D. (2020). Three-dimensional augmented reality robot-assisted partial nephrectomy in case of complex tumours (PADUA ≥10): a new intraoperative tool overcoming the ultrasound guidance. *European Urology*.

[25] Shirk J. D., Thiel D. D., Wallen E. M. (2019). Effect of 3-dimensional virtual reality models for surgical planning of robotic-assisted partial nephrectomy on surgical outcomes. *JAMA Network Open*.

[26] Antonelli A., Veccia A., Palumbo C. (2019). Holographic reconstructions for preoperative planning before partial nephrectomy: a head-to-head comparison with standard CT scan. *Urologia Internationalis*.

[27] Tuderti G., Brassetti A., Mastroianni R. (2022). Expanding the limits of nephron-sparing surgery: surgical technique and mid-term outcomes of purely off-clamp robotic partial nephrectomy for totally endophytic renal tumors. *International Journal of Urology*.

